# Body mass index and leptin levels in serum and cerebrospinal fluid in relation to delayed cerebral ischemia and outcome after aneurysmal subarachnoid hemorrhage

**DOI:** 10.1007/s10143-021-01541-1

**Published:** 2021-04-17

**Authors:** Michael Veldeman, Miriam Weiss, Tim Philipp Simon, Anke Hoellig, Hans Clusmann, Walid Albanna

**Affiliations:** 1 Department of Neurosurgery, RWTH Aachen University Hospital, Pauwelsstrasse 30, 52074 Aachen, Germany; 2 Department of Intensive Care and Intermediate Care, RWTH Aachen University, Aachen, Germany

**Keywords:** Subarachnoid hemorrhage, Delayed cerebral ischemia, Leptin, Body mass index, Fat metabolism

## Abstract

Aneurysmal subarachnoid hemorrhage (SAH) is associated with a high mortality rate and may leave surviving patients severely disabled. After the initial hemorrhage, clinical outcome is further compromised by the occurrence of delayed cerebral ischemia (DCI). Overweight and obesity have previously been associated with protective effects in the post-bleeding phase. The aim of this study was to assess the effects of a patient’s body mass index (BMI) and leptin levels on the occurrence of DCI, DCI-related cerebral infarction, and clinical outcome. In total, 263 SAH patients were included of which leptin levels were assessed in 24 cases. BMI was recorded along disease severity documented by the Hunt and Hess and modified Fisher scales. The occurrence of clinical or functional DCI (neuromonitoring, CT Perfusion) was assessed. Long-term clinical outcome was documented after 12 months (extended Glasgow outcome scale). A total of 136 (51.7%) patients developed DCI of which 72 (27.4%) developed DCI-related cerebral infarctions. No association between BMI and DCI occurrence (*P* = .410) or better clinical outcome (*P* = .643) was identified. Early leptin concentration in serum (*P* = .258) and CSF (*P* = .159) showed no predictive value in identifying patients at risk of unfavorable outcomes. However, a significant increase of leptin levels in CSF occurred from 326.0 pg/ml IQR 171.9 prior to DCI development to 579.2 pg/ml IQR 211.9 during ongoing DCI (*P* = .049). In our data, no association between obesity and clinical outcome was detected. After DCI development, leptin levels in CSF increased either by an upsurge of active transport or disruption of the blood-CSF barrier. This trial has been registered at ClinicalTrials.gov (NCT02142166) as part of a larger-scale prospective data collection. BioSAB: https://clinicaltrials.gov/ct2/show/NCT02142166

**Supplementary Information:**

The online version contains supplementary material available at 10.1007/s10143-021-01541-1.

## Introduction

Aneurysmal subarachnoid hemorrhage (SAH) remains a devastating disease affecting around 9/100,000 people each year [8]. After SAH, the main predictors of outcome are the initial clinical grades either defined by the Hunt and Hess (H&H) or WFNS scale and the amount of subarachnoid blood load stratified according to the modified Fisher scale (mFisher) [7, 37]. Despite general advancements in critical care management for SAH patients, outcome in those with higher clinical grade or blood load remains poor [9, 29]. The initial aneurysm rupture results in a steep increase of intracranial pressure and a sudden drop in cerebral perfusion pressure [30]. It is currently assumed that this initial increase in intracranial pressure and consecutive drop in cerebral perfusion pressure has irreversibly initiated a deleterious cascade coined with the umbrella term early brain injury.

During the first 2 weeks post-hemorrhage, patients remain susceptible to ischemic strokes in which cerebral vasospasm plays an undisputed role alongside many other contributing factors [38]. This delayed cerebral ischemia (DCI) can eventually result in cerebral infarctions, further compromising long-term clinical outcome [39].

Obesity is an established risk factor for cardio- and cerebrovascular disease, surgical complications, and nosocomial infections [2]. Contrary to this association, there is an “obesity paradox” where an increased body mass index (BMI) was associated with an overall lower mortality and complication rate, after ischemic stroke [22, 34] and intracerebral hemorrhage [5, 21]. SAH patients suffering from obesity do not fare worse or may even profit from obesity by unknown mechanisms in regard to DCI development, clinical outcome, and overall rate of complications [6, 28, 36]. A single trial in SAH patients described a lower risk of DCI and DCI-related infarction associated with elevated BMI [33]. Nonetheless, the results of a systematic review addressing the obesity paradox in SAH remained inconclusive as most trials suffered restrictions in design, resulting in limited external validity [31]. In addition, previous studies have exclusively relied on BMI as an obesity variable, even though its sensitivity for high body fat mass has been shown to be only 50% [26].

Leptin, initially discovered as a regulator of food intake and energy expenditure, is emerging as a pleiotropic molecule involved in various physiological and pathological conditions [13, 15]. Under normal physiological circumstances, this peptide has an inhibitory effect on appetite via its modulation of the hypothalamic satiety center. Leptin is, however, also part of a broader neuronal circuit regulating weight and governing energy homeostasis. Crossing the blood–brain barrier, leptin acts on receptors within the central nervous system and exerts an anti-apoptotic effect, increases neuronal survival, and can induce neurogenesis as well as angiogenesis [24]. As part of the cytokine superfamily, leptin has structural and functional similarities with pro-inflammatory cytokines such as interleukin-1, -6, and -12, hence the name adipokine. Furthermore, the leptin receptor (OB-R) is related to class I cytokine receptors, including a common signal-transducing component from the IL-6-related family of cytokines [3]. In obesity, leptin resistance develops, leading to an inability to detect satiety despite sufficient available energy stores. Serum leptin concentrations correlate positively with the percentage of body fat, illustrating the insensitivity of most people suffering from obesity, to endogenous leptin production [4]. This makes leptin an interesting potential obesity variable for assessing the obesity paradox in SAH patients. In the present study, we therefore set out to investigate the role of body fat content for DCI occurrence and long-term outcome after SAH in a prospective manner and based not only on BMI but also on the levels of leptin in cerebrospinal fluid (CSF) and serum. The hypothesis under which this study was designed was that if the obesity paradox holds true, and leptin is an easily available marker for total body fat mass, its concentrations should be inversely related to the risk of DCI and unfavorable outcomes.

## Methods

### Patient population and study design

Patients with aneurysmal subarachnoid hemorrhage presenting at our institution between 2010 and 2018 were screened for eligibility. This observational data collection was approved by the local ethics committee (EK 062/14) and the trial was retrospectively registered (NCT02142166). This manuscript is composed and written according to the STROBE statement for reporting of observational studies. Patients were included in case of confirmed aneurysm rupture on CT or conventional angiography, aged ≥ 18 years. Patients were excluded in case of foreseeable early mortality due to direct brain stem injury (on imaging or uni-/bilateral fixed pupils). Basic demographic data alongside BMI were recorded for each patient on admission based on weight (kilograms) and height (meters). BMI was categorized according the WHO criteria [42] with the addition of an obesity subdivision resulting in five categories: underweight (< 18.5 kg/m^2^), normal weight (≥ 18.5 to < 25 kg/m^2^), overweight (≥ 25 to < 30 kg/m^2^), obesity grade 1 (≥ 30 to < 35 kg/m^2^), and obesity grade 2 (≥ 35 kg/m^2^). This subdivision was added to allow a more precise risk stratification. Initial clinical grade based on the Hunt and Hess grading scale and the amount of subarachnoid blood on CT scanning according to the modified Fisher scale were noted. Additionally, the length of ICU stay and of ventilation was documented for each patient.

Cerebral infarctions diagnosed during DCI (as defined below) or as the first sign of ongoing DCI were registered as DCI-related infarction. Embolic stroke due to halted anticoagulation in patients with cardiac arrhythmias or as a result of surgical or endovascular aneurysm treatment or endovascular DCI rescue treatment was identified based on history and presentation on imaging and excluded. Cases in which multiple DCI-related infarctions triggered withdrawal of technical life-support were labeled as DCI-related mortality. Clinical outcome was assessed by the extended Glasgow Outcome Scale (GOSE) after 12 months by an independent assessor via a structured telephone interview with the patient, his or her next-of-kin or caretaker. Patients were excluded in case of missing long-term outcome data.

### Patient management

All patients were treated according to a standardized treatment protocol consistent over the inclusion period. [1, 43] Aneurysms were secured via endovascular coiling or surgical clipping within 48 h after admission, after which patients were monitored in a neurosurgical intensive care unit. All patients were routinely treated with oral nimodipine in a 6 × 60 mg/day dose. DCI occurrence was either determined by clinical deterioration not attributable to other causes (i.e., hydrocephalus, electrolyte imbalance, seizure, and infection) [40], or functional deterioration defined by a new CT perfusion deficit. Oxygenation (p_ti_O_2_ < 10 mmHg or ≥ 10 mmHg with continuous decrease) or metabolic crisis (lactate/pyruvate ratio ≥ 40 or < 40 with continuous increase) as measured by invasive neuromonitoring probes (Neurovent PTO®, Raumedic®, Helmbrechts, Germany, and 71 High Cut-Off Brain Microdialysis Catheter, μdialysis®, Stockholm, Sweden) were indicators to perform CT perfusion imaging. First-tier treatment was initiated after diagnosis of DCI and consisted of induced euvolemic arterial hypertension (≥ 180 mmHg) by means of intravenous noradrenaline infusion. In refractory cases, second-tier endovascular rescue treatment was considered by either transluminal balloon-angioplasty or continuous intra-arterial spasmolysis [43]. Nutrition was administered according to the guidelines by the European Society for Clinical Nutrition and Metabolism (ESPEN) for ICU patients [35].

### Data collection and subgroup analyses

From patients treated between 2014 and 2016, a sample subgroup of 24 patients was selected and included to be representative for the entire cohort based on age, gender, H&H, and mFisher, with the added prerequisite of having an external ventricular drainage (EVD) in place for cerebrospinal fluid (CSF) collection. Leptin levels were determined in serum and CSF samples during three a priori defined sampling periods: early (d_0-3_), pre-DCI (measurement 2–4 days prior to DCI diagnosis), and during ongoing DCI. Systemic (ng/ml) and CSF leptin (pg/ml) levels were collected once in every epoch, for each individual patient, resulting in three sample pairs (serum and CSF) per patient. Sampling was done in the afternoon (between 12:00 and 17:00) together with routine blood drawing and leptin concentrations were measured in CSF and plasma by means of a sandwich enzyme-linked immune-assay (Human Leptin Kit, Meso Scale Discovery®, Rockville, USA).

Pairs of patient subgroups were defined based on dichotomization of the initial Hunt and Hess clinical grading (good grade H&H_1-2_ *vs.* poor grade H&H_3-5_), modified Fisher scale (mFisher_1-2_ *vs.* mFisher_3-4_), and clinical outcome (unfavorable: GOSE_1-4_ *vs.* favorable: GOSE_5-8_). Additional subgroups were created based on the occurrence of DCI (noDCI *vs.* DCI) and DCI-related cerebral infarction (no infarction *vs.* infarction). An analysis of leptin levels before, during, and after DCI together with the ratio of leptin CSF to plasma concentration, was conducted.

### Outcome definition

The primary endpoint was defined as the outcome assessed by the extended Glasgow outcome scale (GOSE) after 12 months. The GOSE was dichotomized as conventional, into unfavorable (GOSE_1-4_) and favorable (GOSE_5-8_) outcome. Additional outcome parameters were the association between BMI or leptin levels and DCI occurrence, the occurrence of refractory DCI (necessitating endovascular treatment), DCI-related infarction, DCI-related mortality, and overall mortality.

### Statistical analysis

Descriptive statistics are represented as mean and standard deviation for continuous variables unless non-normally distributed in which case median and interquartile range is provided. Categorical data is depicted as frequencies and percentages. Normality testing was done via plotting and the Shapiro–Wilk test. Parametric continuous data were tested using *T*-tests and for non-parametric variables using the Mann–Whitney *U* test. Paired continuous non-parametric data were analyzed via the Wilcoxon signed-rank test, and categorical data were compared using Pearson’s *χ*^2^ test. To assess the predictive value of leptin levels on DCI and outcome, a receiver operating characteristics (ROC) analysis with calculation of the area under the curve (AUC) was performed. To ascertain the correlation between BMI and leptin levels in both compartments in this cohort, the Spearman correlation coefficient was calculated. The alpha significant level was set at a two-sided *P* value below .05, and all statistical analyses were performed using SPSS v. 25 (IBM, Chicago, IL). Illustrations were created with GraphPad Prism 8 (GraphPad Software, Inc., La Jolla, CA, USA).

## Results

### Participants

**Fig. 1:**
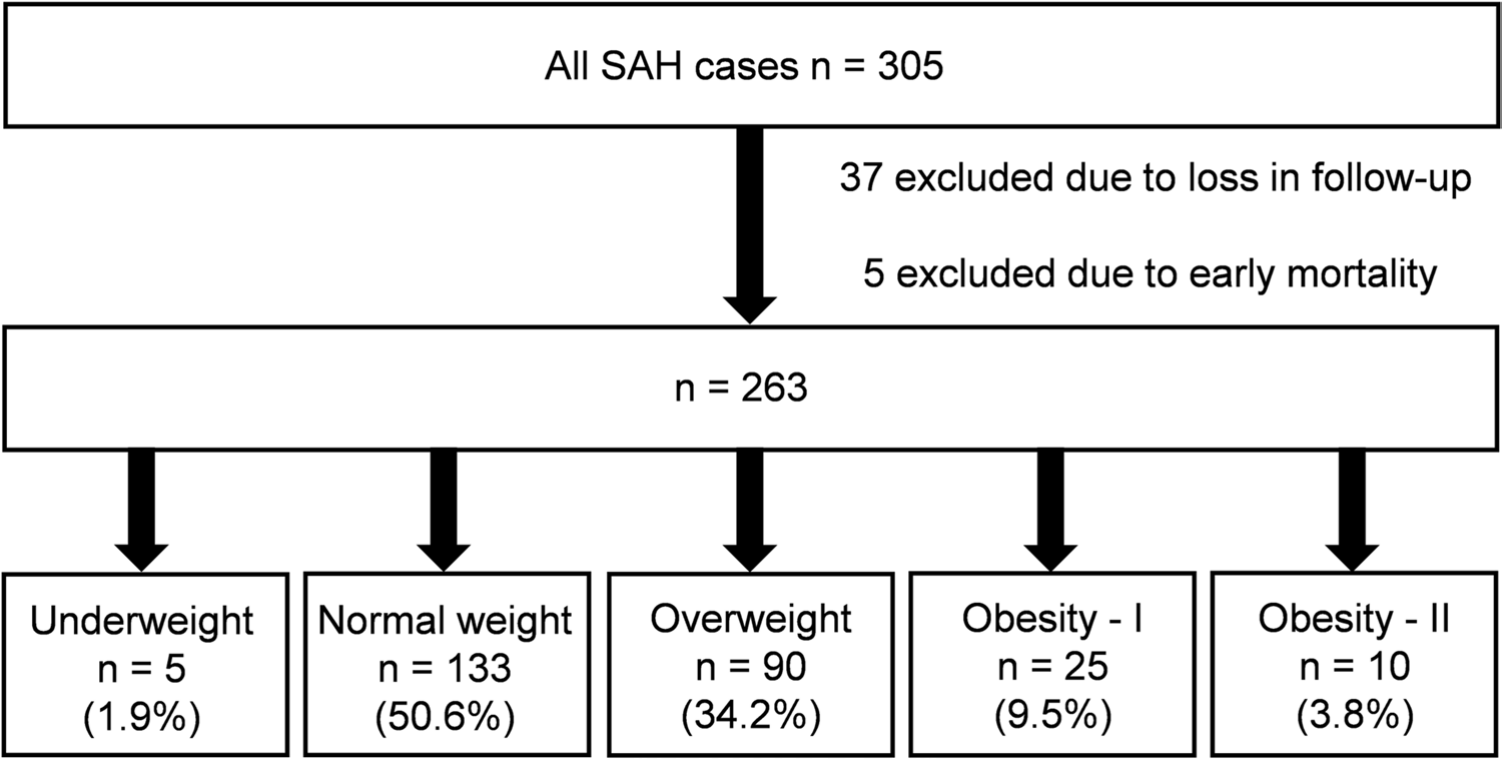
Flowchart of patient recruitment. BMI was categorized according the WHO criteria with the addition of an obesity subdivision resulting in five categories: underweight (< 18.5 kg/m^2^), normal weight (≥ 18.5 to < 25 kg/m^2^), overweight (≥ 25 to < 30 kg/m^2^), obesity grade 1 (≥ 30 to < 35 kg/m^2^), and obesity grade 2 (≥ 35 kg/m^2^). SAH, subarachnoid hemorrhage

A total of 305 admitted SAH patients were analyzed for inclusion between 2010 and 2018. In 37 cases, long-term clinical outcome was missing due to a loss in follow-up. Five patients were excluded because of relevant prior comorbidity. The recruitment process is illustrated in Fig. 1. The mean age of the remaining 263 patients was 53.9 ± 12.7 years with a female to male ratio of 192 (73.0%) to 71 (27.0%). The aneurysm was secured with surgical clipping in 119 (45.2%) patients and via endovascular access in 141 (53.6%) cases. Three patients received a combination of both occlusion techniques. The median BMI was 24.8 ± 5.4 kg/m^2^. Five patients were underweight (1.9%), 133 presented with a normal weight (50.6%), 90 were overweight (34.2%), 25 were classified as obesity grade 1 (9.5%), and 10 as obesity grade 2 (3.8%). No significant differences in outcome-relevant baseline characteristics such as H&H and mFisher grading or aneurysm treatment modality were observed between BMI categories (Suppl. Table 1).

A total of 136 (51.7%) patients developed DCI of which 72 (27.4%) developed DCI-related cerebral infarctions. DCI was the direct cause of death in 27 (10.3%) patients. A favorable outcome was achieved after 12 months in 162 (61.6%) cases. For the analysis of serum and CSF concentrations of leptin, 24 patients were selected from this cohort. Although small, this subgroup proved for the main outcome relevant baseline characteristics comparable to the entire cohort, apart from age. Patients in the subgroup with serum/CSF leptin analyses were on average 5.4 years older (*P* = 0.018). All relevant baseline characteristics are depicted in Table 1.

**Table 1:** Baseline characteristics of all included patients and of patients with leptin assessment in serum and CSF

No. (%)	All (*n* = 263)	No leptin (*n* = 239)	Leptin (*n* = 24)	*P* value*
Age mean ± SD	53.9 ± 12.7	53.3 ± 12.7	58.7 ± 12.0	**.018**
Female/male	192 (73.0)/ 71 (27.0)	173 (72.4) / 66 (27.6)	19 (79.2) / 5 (20.8)	.811
Hunt and Hess				.185
Grade 1	44 (16.7)	43 (18.0)	1 (4.2)	
Grade 2	61 (23.2)	59 (24.7)	3 (12.5)	
Grade 3	83 (31.6)	73 (30.5)	10 (41.7)	
Grade 4	45 (17.1)	40 (16.7)	5 (20.8)	
Grade 5	30 (11.4)	25 (10.5)	5 (20.8)	
mFisher				.432
Grade 1	63 (24.0)	61 (25.5)	2 (8.3)	
Grade 2	39 (14.8)	35 (14.6)	4 (16.7)	
Grade 3	72 (27.4)	67 (28.0)	5 (20.8)	
Grade 4	89 (33.8)	75 (31.4)	13 (54.2)	
Aneurysm location				.298
Acom	87 (33.1)	79 (33.0)	8 (33.3)	
MCA	81 (30.8)	72 (30.1)	9 (37.5)	
ICA	41 (15.6)	38 (15.9)	3 (12.5)	
Other	54 (20.5)	50 (20.9)	4 (16.7)	
AC/PC	208 (79.1) / 55 (20.9)	191 (79.9) / 48 (20.1)	17 (70.8) / 7 (29.2)	
Aneurysm closure**				.283
clipping/endovascular	119 (45.2) / 141 (53.6)	110 (46.0) / 126 (52.7)	9 (37.5) /15 (62.5)	
Risk factors				
Smoking	78 (29.7)	73 (30.5)	5 (20.8)	.482
Hypertension	105 (40.0)	93 (38.9)	12 (50.0)	.189
DM2	10 (3.8)	8 (3.3)	2 (8.3)	.229
BMI	24.8 ± 5.4	25.7 ± 4.9	25.8 ± 4.3	.855
Overweight	90 (34.2)	80 (33.5)	10 (25.0)	.259
Obesity	35 (13.3)	32 (13.4)	3 (12.5)	.750
DCI incidence	136 (51.7)	122 (51.0)	14 (58.3)	.534
DCI-related infarction	72 (27.4)	66 (27.6)	6 (25.0)	.568
DCI-related mortality	27 (10.3)	25 (10.5)	2 (8.3)	.764
Favorable outcome (GOSE_5-8_)	162 (61.6)	147 (61.5)	15 (62.5)	.931

Significant results of univariate analysis are writen in bold

^*^All statistics are the results of comparing all patients without leptin measurements (*n* = 239) versus those with leptin measurements (*n* = 24)

^**^Three patients were treated with a combination of endovascular occlusion and surgical clipping

*Acom*, anterior communicating artery; *AC/PC*, anterior circulation / posterior circulation; *BMI*, body mass index; *DCI*, delayed cerebral ischemia; *DM2*, type 2 diabetes; *ICA*, internal carotid artery; *MCA*, middle cerebral artery; *mFisher*, modified Fisher grade; *SD*, standard deviation

### BMI, DCI, and outcome

When comparing BMI in subgroups based on DCI occurrence (no DCI = 25.2 kg/m^2^ ± 4.4 *vs.* DCI = 26.0 kg/m^2^ ± 5.1; *P* = .095), DCI-related infarction (no infarction: 25.7 kg/m^2^ ± 4.9 *vs.* infarction: 25.5 kg/m^2^ ± 4.6; *P* = .522), and overall mortality (survivors: 25.6 kg/m^2^ ± 4.8 *vs.* died: 25.7 kg/m^2^ ± 4.9; *P* = .652), no significant differences were identified. Similarly, non-significant results were obtained when comparing patients reaching unfavorable (GOSE_1-4_) or favorable outcome (GOSE_5-8_), and between outcome groups, the mean BMI for both was almost identical (GOSE_1-4_: 24.8 kg/m^2^ ± 5.6 vs. GOSE_5-8_: 24.9 kg/m^2^ ± 5.2; *P* = .995) (Table 2). The incidence of DCI varied between BMI categories between 40.0 and 80.0%. No significant differences were noted between obesity categories regarding incidence of DCI *χ*^2^ (4, 263) = 3.969, *P* = .410; DCI-related infarction *χ*^2^ (4, 263) = 3.473, *P* = .301 or DCI-related mortality *χ*^2^ (4, 263) = 2.983; *P* = .304. Even in the extreme BMI categories, favorable outcome was reached in three out of five underweight patients and in five out of ten severely obese patients (Table 3).

**Table 2:** Results comparing outcome subgroups of dichotomized GOSE after 12 months. In this univariate analysis, only Hunt and Hess and mFisher, not BMI, differed between outcome subgroups

	Unfavorable outcome	Favorable outcome	
Variables (*n* = 263)	GOSE_1-4_	GOSE_5-8_	
No. (%)	*n* = 101	*n* = 162	*P* value
BMI, mean ± SD	24.8 (5.6)	24.9 (5.2)	.995
Dichotomized—BMI			.800
< 25 kg/m^2^	53 (52.5)	85 (52.5)	
≥ 25 kg/m^2^	48 (47.5)	77 (47.5)	
Categorized—BMI			.258
< 18.5	2 (2.0)	3 (1.9)	
≥ 18.5; < 25	51 (50.5)	82 (50.6)	
≥ 25; < 30	29 (28.7)	61 (37.7)	
≥ 30; < 35	14 (13.9)	11 (6.8)	
BMI ≥ 35	5 (5.0)	5 (3.1)	
Hunt and Hess			** < .0001**
Grade 1	5 (5.0)	39 (24.1)	
Grade 2	7 (6.9)	54 (33.3)	
Grade 3	32 (31.7)	51 (31.5)	
Grade 4	33 (32.7)	12 (7.4)	
Grade 5	24 (23.8)	6 (3.7)	
mFisher			** < .0001**
Grade 1	6 (5.9)	57 (35.2)	
Grade 2	5 (5.0)	34 (21.0)	
Grade 3	38 (37.6)	34 (21.0)	
Grade 4	52 (51.5)	37 (22.8)	
Aneurysm closure			.537
Clipping/endovascular	43 (42.6) / 57 (56.4)*	76 (46.9) / 86 (53.1)	

Significant results of univariate analysis are writen in bold

^*^Three patients were treated with a combination of endovascular occlusion and surgical clipping

*BMI*, body mass index; *GOSE*, extended Glasgow outcome scale; *mFisher*, modified Fisher scale; *SD*, standard deviation

**Table 3:** Results comparing clinical outcome and complications between BMI categories

		Underweight	Normal weight	Overweight	Obesity—I	Obesity—II	
Variables (*n* = 263)	All	BMI < 18.5	BMI ≥ 18.5; < 25	BMI ≥ 25; < 30	BMI ≥ 30; < 35	BMI ≥ 35	
No. (%)	*n* = 263	*n* = 5	*n* = 133	*n* = 90	*n* = 25	*n* = 10	*P* value
DCI	136 (51.7)	2 (40)	68 (51.1)	44 (48.9)	14 (56.0)	8 (80.0)	.410
Refractory DCI	65 (24.7)	2 (40)	33 (24.8)	18 (20.0)	9 (36.0)	3 (30.0)	.564
DCI-related infarction	72 (27.4)	1 (20)	38 (28.6)	20 (22.2)	10 (40.0)	3 (30.0)	.301
Mortality							
DCI-related	27 (10.3)	1 (20)	14 (10.5)	7 (7.8)	5 (20.0)	0 (0.0)	.304
Complications							
All infections	102 (38.8)	2 (40.0)	52 (39.1)	29 (32.2)	12 (48.0)	7 (70.0)	.200
Pneumonia	102 (38.8)	2 (40.0)	49 (36.8)	33 (36.7)	12 (48.0)	7 (70.0)	.314
Sepsis	40 (15.2)	0 (0.0)	24 (18.0)	10 (7.5)	4 (16.0)	2 (20.0)	.647
UTI	25 (9.5)	0 (0.0)	10 (7.5)	11 (12.2)	4 (16.0)	0 (0.0)	.267
ICP crises	66 (25.1)	1 (20.0)	28 (21.1)	25 (27.8)	8 (32.0)	4 (40.0)	.667
DHC	52 (19.8)	1 (20.0)	24 (18.0)	19 (21.1)	4 (16.0)	4 (40.0)	.197
Outcome—GOSE 12 *mo*							.643
Dead	55 (20.9)	1 (20)	29 (21.8)	15 (16.7)	9 (36.0)	1 (10.0)	
Vegetative state	10 (3.8)	1 (20)	4 (3.0)	2 (2.2)	2 (8.0)	1 (10.0)	
Lower severe disability	13 (4.9)	0 (0.0)	5 (3.8)	5 (5.6)	2 (8.0)	1 (10.0)	
Upper severe disability	24 (9.1)	0 (0.0)	13 (9.8)	8 (8.9)	1 (4.0)	2 (20.0)	
Lower moderate disability	27 (10.3)	0 (0.0)	14 (10.5)	10 (11.1)	2 (8.0)	1 (10.0)	
Upper moderate disability	33 (12.5)	1 (20)	12 (9.0)	17 (18.9)	2 (8.0)	1 (10.0)	
Lower good recovery	43 (16.3)	0 (0.0)	22 (16.5)	16 (17.8)	2 (8.0)	3 (30.0)	
Upper good recovery	58 (22.1)	2 (40)	34 (25.6)	17 (18.9)	5 (20.0)	0 (0.0)	
Favorable outcome							
GOSE_4-8_	161 (61.2)	3 (60.0)	82 (67.8)	60 (66.7)	11 (44.0)	5 (50.0)	.227
Relative risk (95% CI)		1.031 (0.501—2.123)	0.998 (0.825—1.208)	1.012 (0.827—1.239)	0.959 (0.702—1.308)	1.028 (0.614—1.720)	
Unfavorable outcome							.227
GOSE_1-5_	102 (38.8)	2 (40.0)	51 (38.3)	30 (33.3)	14 (56.0)	5 (50.0)	
Relative risk (95% CI)		0.953 (0.322—2.821)	1.003 (0.739—1.362)	0.981 (0.712—1.352)	1.074 (0.622—1.855)	0.958 (0.442 to- 2.081)	

*DCI*, delayed cerebral ischemia; *DHC*, decompressive hemicraniectomy; *GOSE*, extended Glasgow outcome scale; *ICP*, intracranial pressure; *UTI*, urinary tract infection

### Early leptin levels

There was a statistically significant positive correlation between early serum leptin levels and BMI, (r_s_(22) = 0.503; *P* = .012) as well as early CSF leptin levels and BMI (*r*_s_(22) = 0.766; *P* < .001). In a ROC analysis, early leptin concentration in serum (AUC = 0.662; 95% CI: 0.378 to 0.947, *P* = .258) and CSF (AUC = 0.712; 95% CI: 0.454 to 0.971; *P* = .159) showed no predictive value in identifying patients at risk of unfavorable outcome. Similarly, early leptin levels in serum (AUC = 0.688; 95% CI: 0.419 to 0.956; *P* = .137) and CSF (AUC = 0.571; 95% CI: 0.263 to 0.880; *P* = .626) were not associated with later DCI occurrence.

### Leptin levels around DCI

**Fig. 2:**
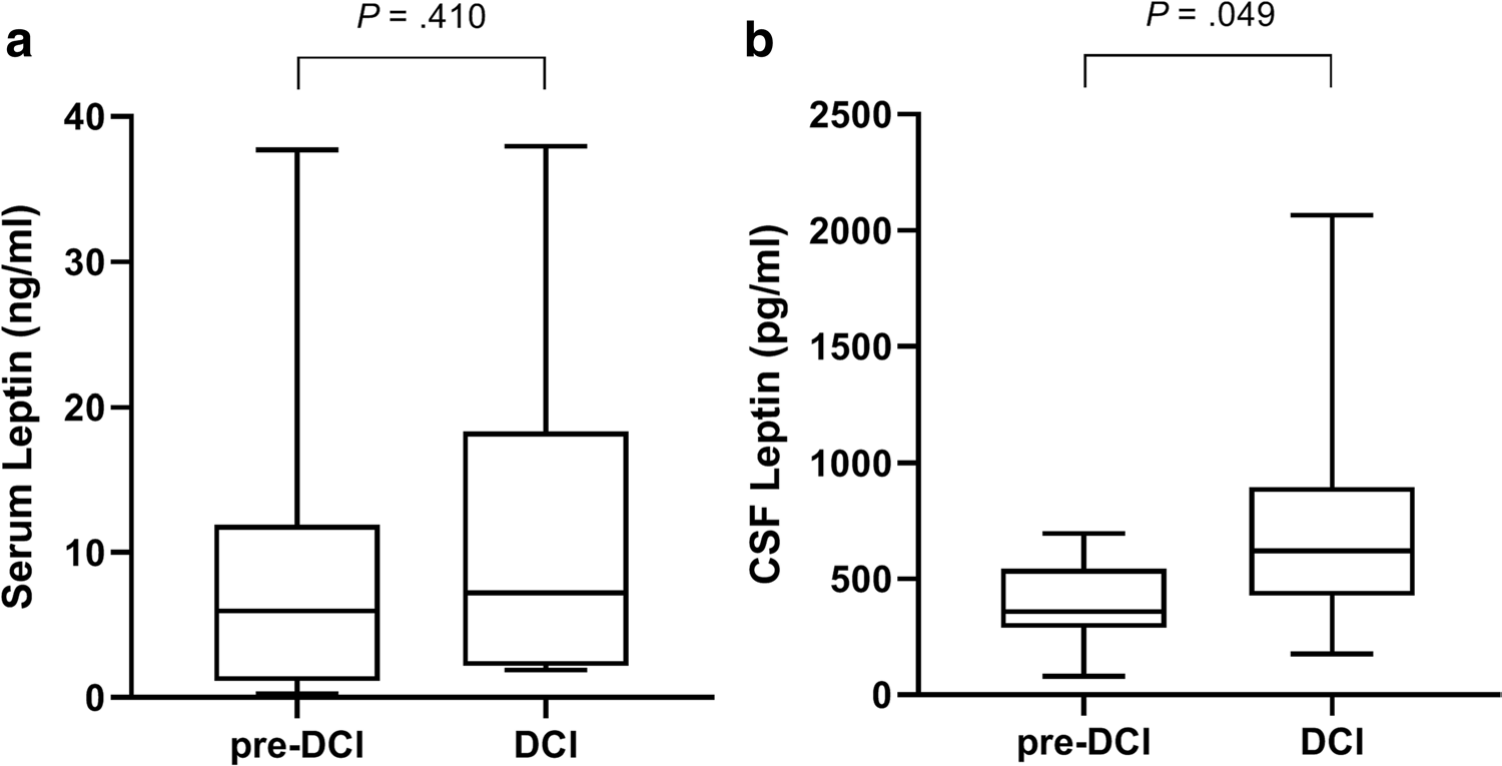
Comparison of leptin levels in serum (**a**) and CSF (**b**) before and after the occurrence of DCI (*n* = 14). As pre-DCI, leptin measurements available between 2 and 4 days prior to the initial DCI diagnosis were used. A statistically significant increase of leptin levels in CSF from 326.0 pg/ml IQR 171.90 to 579.2 pg/ml IQR 211.9 (*P* = .049) was observed. CSF, cerebrospinal fluid; DCI, delayed cerebral ischemia; pre-DCI, measurement 2–4 days prior to DCI diagnosis

Of the 14 patients who developed DCI, leptin levels in serum were compared before and during DCI occurrence. There was a non-significant increase of systemic concentrations in patients developing DCI from a median level of 5.97 ng/ml IQR 11.4 to 7.22 IQR 16.3 ng/ml (*P* = .410). In CSF, a statistically significant increase of leptin levels from 326.0 pg/ml IQR 171.90 to 579.2 pg/ml IQR 211.9 (*P* = .049) was observed. The comparison of both time points is depicted in Fig. 2.

## Discussion

This observational study was conducted to assess whether obesity, presented by a higher BMI and higher systemic leptin levels, is protective against DCI and results in better clinical outcomes. Our results illustrate no such association neither between BMI—either as a continuous or categorized variable—nor leptin concentrations, with DCI or clinical outcome.

The obesity paradox concerning clinical outcome in SAH emerged on the basis of oftentimes inconclusive studies. The two largest studies were based on patient registries yielding considerable case numbers but came to diverging results with one trial documenting reduced in-hospital mortality and the other rejecting the obesity paradox [6, 10]. The methodological integrity of these trials was critically addressed in a recent systematic review [31]. The majority of trials so far have focused on early in-hospital mortality independent of the cause or relation to DCI. In this trial, a rigorous clinical and multimodal definition was applied surpassing mere angiographic vasospasm, as in previous studies. Our results did not identify a beneficial effect of higher body fat mass against DCI occurrence. It remains however conceivable that a protective effect of mild obesity exists—unspecific to SAH or stroke—potentially related to overall caloric reserves which may prove advantageous in the light of long ICU stays [21, 27]. For example, overweight defined by a body mass index between 25 and 30 is associated with lower mortality rates in patients with severe sepsis [41]. This effect was however not detected in our cohort.

A positive influence of obesity on the occurrence of DCI and hereby caused cerebral infarction was however observed in prior research. Rinaldo et al. reported a significant reduction of DCI in patients with higher BMI and postulated a possible neuroprotective effect of leptin [33]. These data served as an incentive to substantiate the correlation of BMI and DCI with systemic and CSF levels of leptin as the possible mediator of the proposed positive effect of obesity [32]. Although a control group with healthy subjects was not available, measured leptin concentration in SAH patients transcended, in any of the evaluated time frames, the proposed normal reference range [14]. To our knowledge, there have been four previous trials assessing human leptin levels after SAH, all focusing on samples taken during the first post-hemorrhage days [11, 16, 23, 25]. All trials consistently demonstrated higher baseline leptin serum levels compared to healthy individuals. Fan et al. were the first to assess serum leptin levels in SAH patients in comparison to healthy volunteers [11]. Leptin levels proved higher in patients who suffered SAH, and the height of leptin concentrations was positively correlated with the severity of disease as measured by the WFNS scale. In another observational study investigating 96 women, leptin levels remained independent of the initial clinical grade as measured by the H&H scale but gender inequalities in leptin levels may have led to bias in this investigation [16]. After SAH, serum leptin levels increase rapidly to peak after 24 h. Thereafter, the serum concentrations decrease gradually but remain substantially higher compared to healthy controls during the following 7 days [25]. This further illustrates the systemic nature of SAH as a disease where multiple organ systems become involved. However, leptin levels were not predictive of DCI or clinical outcome in our cohort.

Notably, DCI itself might affect the concentrations of leptin in CSF, which were higher in patients after onset of DCI. As there are no indications of leptin production within the central nervous system, CSF levels are expected to be the result of transport across the blood–brain or blood-CSF barrier. Increased levels during DCI may reflect a passive accumulation of leptin mediated by ischemic injury to the blood-CSF barrier. As leptin has been proposed to also possess an immunomodulatory function, some role of leptin in the progression or internal compensatory mechanisms within the inflammatory cascade initiate by ischemic injury cannot be excluded but would certainly warrant further studies once the complex DCI cascade is better understood [3, 4, 18].

### Limitations

In common with most clinical SAH studies, the most important limitation of our study is the small number of patients that could be recruited. Thus, even though the sample size was comparable to or even exceeds that of many previous studies published in this context [17, 19, 20, 33], the deleterious effect of underweight or severe obesity might have been underestimated due to the limited number of patients in these categories and caution is warranted to extrapolate our results to more extreme BMI values. Even more importantly, the small overall number of patients means that, like in most previous studies, our analysis was notably underpowered and thus unable to detect small effects of obesity. Our findings should therefore only be regarded as evidence for the lack of a strong association between BMI or leptin levels and the development of DCI. As exemplified in a recent systematic review [31], a much larger number of patients in all obesity categories would be required to detect more subtle protective effects of body fat mass in SAH patients. Also, leptin is a hormone susceptible to diurnal variations related to feelings of hunger and satiety. These fluctuations have not been described in ICU patients and our results are based on a single daily measurement. A further limitation is the handpicked inclusion of patients in which leptin levels were assessed. As this was not performed at random, a selection bias might be present even if its character is unknown. The sample was selected to reflect the baseline characteristics of the entire cohort, but the number of patients remains fairly small increasing the chance of an error of the second kind. The difference identified between leptin levels before and after DCI was only marginally significant and, taking into account that we did not correct for the increased error probability due to multiple comparisons, is unlikely to represent a truly significant difference. Finally, as with other regulators of the inflammatory response, leptin function may be modulated by local leptin concentration, the ratio between free and bound leptin, the expression of different forms of the receptors, the ratio between signaling and non-signaling receptors, and the presence of specific inhibitors [12].

## Conclusion

In this analysis, obesity or increased BMI was not associated with a reduced rate of DCI and better clinical outcomes. Based on these results, obesity seems not to play a relevant role in producing better or worse clinical outcomes. Similarly, early leptin levels do not differ between patients developing DCI or not. After developing DCI, leptin levels in CSF increase either by active transport or disruption of the blood-CSF barrier.

## Supplementary Information

Below is the link to the electronic supplementary material.

Supplementary Table 1 Comparison of baseline characteristics between BMI categories. AC = anterior circulation; BMI = body mass index; DM2 = type 2 diabetes; mFisher = modified Fisher scale; PC = posterior circulation; SD = standard deviation. *Three patients were treated with a combination of endovascular occlusion and surgical clipping. (DOCX 15 KB)

## Data Availability

The raw data of this analysis can be made available by the authors to any qualified researcher.
