# Supplementary material for: Body mass index and leptin levels in serum and cerebrospinal fluid in relation to delayed cerebral ischemia and outcome after aneurysmal subarachnoid hemorrhage
Source: Neurosurg Rev. 2021 Apr 17;44(6):3547–56. doi: 10.1007/s10143-021-01541-1 (PMC8593057; doi:10.1007/s10143-021-01541-1)
Supplement: Supplementary file 1 — Comparison of baseline characteristics between BMI categories. AC = anterior circulation; BMI = body mass index; DM2 = type 2 diabetes; mFisher = modified Fisher scale; PC = posterior circulation; SD = standard deviation. *Three patients were treated with a combination of endovascular occlusion and surgical clipping. (DOCX 15 KB) [file 10143_2021_1541_MOESM1_ESM.docx]

|  |  | Underweight | Normal weight | Overweight | Obesity - I | Obesity Grade - II |  |
| --- | --- | --- | --- | --- | --- | --- | --- |
| Variables (n = 263) | All | BMI < 18.5 | BMI ≥ 18.5; < 25 | BMI ≥ 25; < 30 | BMI ≥ 30; < 35 | BMI ≥ 35 |  |
| No. (%) | n = 263 | n = 5 | n = 133 | n = 90 | n = 25 | n = 10 | P-value |
| Age, mean ± SD | 53.9 ± 12.7 | 51.0 ± 5.9 | 53.3 ± 1.1 | 54.1 ± 1.3 | 58.0 ± 2.8 | 50.0 ±3.3 | .427 |
| Female / Male | 192 (73.0) / 71 (27.0) | 3 (60.0) / 2 (40.0) | 97 (72.9) / 36 (27.1) | 65 (72.2) / 25 (27.8) | 20 (80.0) / 5 (20.0) | 7 (70.0) / 3 (30.0) | .890 |
| Hunt & Hess |  |  |  |  |  |  | .896 |
| Grade 1 | 44 (16.7) | 0 (0.0) | 25 (18.8) | 12 (13.3) | 6 (24.0) | 1 (10.0) |  |
| Grade 2 | 61 (23.2) | 2 (40.0) | 34 (25.6) | 17 (18.9) | 5 (20.0) | 3 (30.0) |  |
| Grade 3 | 83 (31.6) | 2 (40.0) | 37 (27.8) | 33 (36.7) | 7 (28.0) | 4 (40.0) |  |
| Grade 4 | 45 (17.1) | 0 (0.0) | 23 (17.3) | 16 (17.8) | 4 (16.0) | 2 (20.0) |  |
| Grade 5 | 30 (11.4) | 1 (20.0) | 14 (10.5) | 12 (13.3) | 3 (12.0) | 0 (0.0) |  |
| mFisher |  |  |  |  |  |  | .179 |
| Grade 1 | 63 (24.0) | 1 (20.0) | 29 (21.8) | 24 (26.7) | 7 (28.0) | 2 (20.0) |  |
| Grade 2 | 39 (14.8) | 0 (0.0) | 24 (18.0) | 11 (12.2) | 4 (16.0) | 0 (0.0) |  |
| Grade 3 | 72 (27.4) | 1 (20.0) | 42 (31.6) | 17 (18.9) | 6 (24.0) | 6 (60.0) |  |
| Grade 4 | 89 (33.8) | 3 (60.0) | 38 (28.6) | 38 (42.2) | 8 (32.0) | 2 (20.0) |  |
| Aneurysm location |  |  |  |  |  |  | .351 |
| AC / PC | 208 (79.1) / 55 (20.9) | 4 (80.0) / 1 (20.0) | 105 (78.9) / 28 (21.1) | 75 (83.3) / 15 (16.7) | 16 (64.0)/ 9 (36.0) | 8 (80.0) / 2 (20.0) |  |
| Aneurysm closure |  |  |  |  |  |  | .838 |
| clipping / endovascular | 119 (45.2) / 141 (53.6)* | 1 (20.0) / 4 (80.0) | 61 ( 45.9) / 72 (54.1) | 41 (45.6) / 48 (53.3) | 11 (44.0) / 14 (56.0) | 5 (50.0) / 5 (50.0) |  |
| Risk factors |  |  |  |  |  |  |  |
| Smoking | 78 (29.7) | 1 (20.0) | 37 (27.8) | 27 (30.0) | 7 (28.0) | 6 (60.0) | .299 |
| Hypertension | 105 (39.9) | 2 (40.0) | 53 (39.8) | 30 (33.3) | 15 (60.0) | 5 (50.0) | .181 |
| DM2 | 10 (3.8) | 0 (0.0) | 5 (3.8) | 1 (1.1) | 3 (12.0) | 1 (10.0) | .106 |

**Supplementary table 1**
